# Network-Based Approaches to Explore Complex Biological Systems towards Network Medicine

**DOI:** 10.3390/genes9090437

**Published:** 2018-08-31

**Authors:** Giulia Fiscon, Federica Conte, Lorenzo Farina, Paola Paci

**Affiliations:** 1 Institute for Systems Analysis and Computer Science “Antonio Ruberti”, National Research Council, via dei Taurini 19, 00185 Rome, Italy; giulia.fiscon@iasi.cnr.it (G.F.); federica.conte@iasi.cnr.it (F.C.); 2 SysBio Centre of Systems Biology, Piazza della Scienza, 3, 20126 Milan, Italy; 3 Department of Computer, Control, and Management Engineering “Antonio Ruberti”, Sapienza University of Rome, Viale Ariosto 25, 00185 Rome, Italy; farina@diag.uniroma1.it

**Keywords:** bioinformatics, network medicine, gene co-expression network, regulatory network, ceRNA, PPI network

## Abstract

Network medicine relies on different types of networks: from the molecular level of protein–protein interactions to gene regulatory network and correlation studies of gene expression. Among network approaches based on the analysis of the topological properties of protein–protein interaction (PPI) networks, we discuss the widespread DIAMOnD (disease module detection) algorithm. Starting from the assumption that PPI networks can be viewed as maps where diseases can be identified with localized perturbation within a specific neighborhood (i.e., disease modules), DIAMOnD performs a systematic analysis of the human PPI network to uncover new disease-associated genes by exploiting the connectivity significance instead of connection density. The past few years have witnessed the increasing interest in understanding the molecular mechanism of post-transcriptional regulation with a special emphasis on non-coding RNAs since they are emerging as key regulators of many cellular processes in both physiological and pathological states. Recent findings show that coding genes are not the only targets that microRNAs interact with. In fact, there is a pool of different RNAs—including long non-coding RNAs (lncRNAs) —competing with each other to attract microRNAs for interactions, thus acting as competing endogenous RNAs (ceRNAs). The framework of regulatory networks provides a powerful tool to gather new insights into ceRNA regulatory mechanisms. Here, we describe a data-driven model recently developed to explore the lncRNA-associated ceRNA activity in breast invasive carcinoma. On the other hand, a very promising example of the co-expression network is the one implemented by the software SWIM (switch miner), which combines topological properties of correlation networks with gene expression data in order to identify a small pool of genes—called switch genes—critically associated with drastic changes in cell phenotype. Here, we describe SWIM tool along with its applications to cancer research and compare its predictions with DIAMOnD disease genes.

## 1. Network Medicine: An Emergent Paradigm in Medicine

The exploitation of the emerging network-based approaches to medicine enables multiple potential biological and clinical applications by offering an intuitive and reliable way to explore systematically the molecular complexity of a particular disease and thus leading to the identification of disease genes as potential drug targets and biomarkers. A disease is rarely a consequence of an abnormality in a single gene, but reflects the perturbations of the complex network of intracellular and intercellular interactions. This entirely new perspective, in which critical biological factors are nearly always the result of multiple pathobiological pathways that interact through an interconnected network to control disease pathobiology, has been able to kick-start a new medical paradigm called “Network Medicine” [1]. The fundamental tenet of network medicine is to look at diseases as perturbations within the interactome, i.e., the comprehensive network map of molecular components and their interactions [1,2]. The overall ambition is both to develop a global understanding of how interactome perturbations result in disease traits, and to translate computational insights into concrete clinical applications, such as new drugs and therapies or diagnostic tools. Furthermore, the representation of biological complex systems as networks enables the visualization of the interactome underlying structure, revealing new functional roles, and proposing new and fresh interpretations of data.

**Figure 1:**
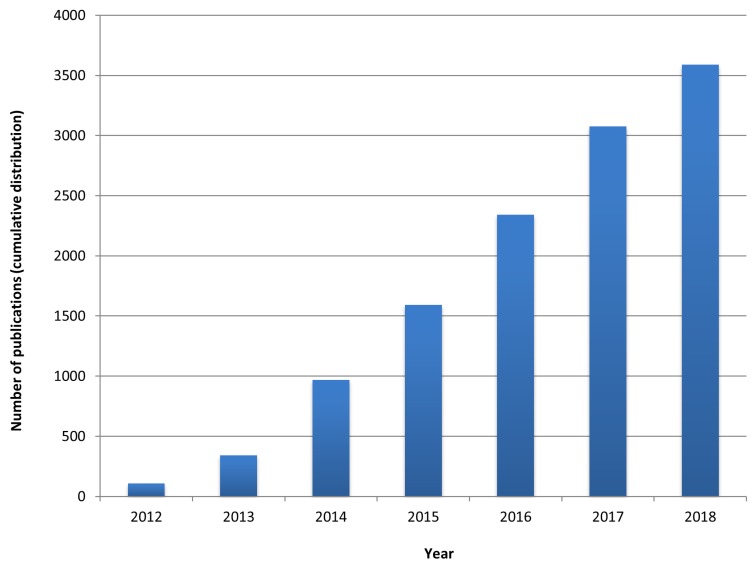
Number of 2012–2018 publications related to network-based approaches to medicine. The figure shows the number of articles published by year obtained by searching the following specific keywords in Pubmed: network-based approach, network medicine, biological network.

A network is a set of nodes and edges, where nodes are linked together if a kind of interaction occurs between them. As witnessed by the last-years increasing number of publications (Figure 1), most attention has been recently directed towards molecular networks, including:

1. Protein–protein interaction (PPI) networks, whose nodes are proteins that are linked to each other by physical interactions [3,4];
2. Regulatory networks, whose directed links represent regulatory relationships between a transcription factor and a gene [5];
3. Co-expression networks, in which genes with similar co-expression patterns are linked [6].

Table 1 summarizes some of the widespread disease-gene prediction methods/tools based on the analysis of the topological properties of the above-mentioned molecular networks.

The PPI-network based approaches can be mainly classified into: (i) local methods, based on searching for direct interactions between candidate genes and known disease genes (e.g., Oti et al. [7], GenePANDA [8]); (ii) global methods, that model how the information flow in the cell to assess the proximity and connectivity between known disease genes and candidate genes (e.g., DADA [9], DIAMOnD [10], PRINCE [11], ProDiGe [12]). In this review, we provide a detailed description of DIAMOnD [10], the most used and newest approach at the state of the art.

In the past, a great effort has been devoted to understanding the molecular mechanism of post-transcriptional regulation with a special emphasis on non-coding RNAs (ncRNAs) since they are emerging as key regulators of many cellular processes in both physiological and pathological states [13,14,15,16]. This class of RNA species appears really heterogeneous, including the intensively studied microRNAs (miRNAs)—small non-coding RNAs of 20–22 nucleotides long—as well as the most recent acknowledged long non-coding RNAs (lncRNAs)—non-protein coding RNAs greater than 200 nucleotides in length and lacking of extended open reading frames [17,18,19]. Recent findings show that coding genes are not the only targets that miRNAs interact with. In fact, there is a pool of different RNAs competing with each other to attract miRNAs for interactions, thus acting as competing endogenous RNAs (ceRNAs). This intriguing mechanism, also known as “target mimicry” process, was first discovered in plants [20]. Crucial triggers of this new layer of post-transcriptional regulation are “decoys”—or miRNA “sponges”—including both coding and non-coding RNAs, such as pseudogenes, lncRNAs, large intergenic ncRNAs, and circular RNAs [21,22,23,24]. Sponges exert their decoy activity by recruiting miRNA molecules via base-pairing with miRNA-recognition elements (MREs), which they share with a target, subsequently causing release of the target from miRNA control. The framework of regulatory networks provides a powerful tool to gather new insights into ceRNA regulatory mechanisms. Among computational methods describing miRNA-sponge interactions by exploiting gene regulatory networks approach, the ceRNA model of Paci et al. [25] stood out as the best method in terms of the percentage of discovered miRNA-sponge interactions associated with breast cancer, according to a thorough and comparison study proposed in [26]. In this review, we provide a detailed description of ceRNA model implemented by Paci et al. [25] along with its application to human breast invasive carcinoma [27].

The framework of co-expression networks provides a powerful tool to gather biologically relevant information from the interaction structure underpinning patterns of gene expression, thus accelerating the interpretation of molecular mechanisms at the root of significant biological processes. A new promising approach based on the co-expression network is the one implemented by SWIM (SWItch Miner) [28], a software able to identify key genes in a network of interactions of various sorts by defining appropriate roles of genes according to their local/global positioning in the overall network. The latter property being of crucial importance, given that, recently, it has been shown that genes associated with a disease are localized in specific neighborhoods, or disease modules, within the interactome [1]. SWIM builds the co-expression network by using the Pearson correlation coefficient between the expression profiles of any pair of genes. Nodes in this network are RNA transcripts and a link occurs between two nodes if their expression profiles are highly correlated or highly anti-correlated. In order to identify disease modules, SWIM makes use of network clustering algorithms like k-means and, then, assigns a role to each node in the network according to its inter- and intra-cluster interaction. Understanding diseases in the context of these networks allows to address some fundamental properties of the disease-specific genes, which are called “switch genes” by SWIM. In this review, we provide a detailed description of SWIM along with its applications in viticulture [29] and oncology [28,30].

Notably, another tool based on the co-expression network is WGCNA (Weighted Correlation Network Analysis) [31], a comprehensive collection of R functions for performing various aspects of gene co-expression network analysis on high-dimensional data, including functions for network construction, module detection, gene selection, calculations of topological properties, data simulation and visualization.

This review is organized as follows: in Section 2, we detail DIAMOnD algorithm developed by Barabasi and co-authors [10] to discover new candidate disease genes associated with a particular phenotype; in Section 3, we detail the ceRNA model proposed by Paci et al. [25,27] and its application to human breast cancer; in Section 4, we describe the software SWIM (SWItch Miner) [28] and its applications in viticulture [29] and oncology [28,30].

## 2. DIseAse MOdule Detection (DIAMOnD)

Recently, several studies have shown how cellular components associated with a specific disease are not randomly scattered within the human interactome, but agglomerate in specific regions, suggesting the existence of specific “disease modules” for each disease. Barabasi et al. [1] proposed a research pipeline for the identification and validation of disease modules. In particular, for any specific disease the pipeline consists of the following steps:

- Interactome reconstruction merges the most up-to-date information on protein–protein interactions, co-complex memberships, regulatory interactions and metabolic network maps in the tissue and cell line of interest.
- Disease gene (seed) identification collects the known disease-associated genes obtained from linkage analysis, genome-wide association studies or other sources, which serve as the seed of the disease module.
- In disease module identification, the seed genes are placed on the interactome, with the aim of identifying a subnetwork that contains most of the disease-associated components, exploiting both the functional and topological modularity of the network.
- Pathway identification can be used in instances in which the number of components contained in the ascertained disease module is so large that it cannot serve as a tractable starting point for further experimental work.
- Disease modules are tested for their functional and dynamic homogeneity.

**Figure 2:**
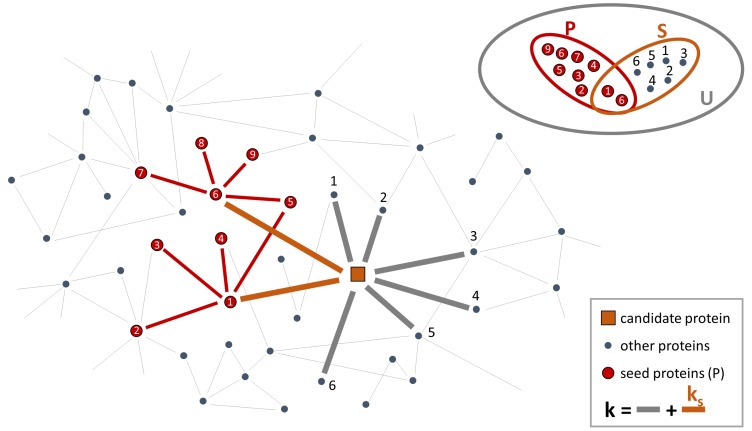
Sketch of step 1 of DIAMOnD. The network corresponds to the interactome where the red balls are the seed proteins, the orange square is the protein to test with *k* connections (orange and grey thick links) including $k_{s}$ links to seed proteins (orange thick links), the grey balls refer to other proteins in the PPI-network. The sets at top-right correspond to: U is the ensemble of the total number of nodes in the PPI-network, S is the ensemble of the draw of *k* proteins, including $k_{s}$ seed proteins ($k_{s}=2$ in this example), P is the ensemble of the seed proteins.

More recently, Barabasi and co-authors proposed a novel algorithm—DIAMOnD (DIseAse MOdule Detection) [10]—to uncover disease modules associated with a particular phenotype. By systematically analyzing the protein–protein interactions of 70 diseases, they showed that disease modules do not coincide with topological communities of densely interconnected proteins and instead identified the interaction significance as the key quantity to characterize the connection patterns among disease proteins. To extract disease modules, DIAMOnD algorithm starts from proteins known to be associated with a particular disease (i.e., seed proteins) and prioritizes the other proteins of the interactome having a significant fraction of their interactions with seed proteins. In particular, DIAMOnD encompasses the following steps:

1. For all proteins with at least one connection to any of the seed proteins, it calculates the “connectivity significance”. Specifically, DIAMOnD uses the hypergeometric distribution to calculate the statistical significance of having drawn $k_{s}$ seed proteins (out of *k* total draws) from a population of *N* proteins including $s_{0}$ seed proteins. The hypergeometric distribution is:
  (1) p(k,ks)=s0ksN−s0k−ksNk
  and then, the “connectivity significance” is obtained as:
  (2) $$p\text{-value}\left(k,k_{s}\right)=\sum_{k_{i}=k_{s}}^{k}p\left(k,k_{i}\right)$$
  In a network view, the population of *N* proteins corresponds to the nodes of the PPI-network and *k* are the nearest neighbors of a certain protein in the network. This set of nearest neighbors must include $k_{s}$ seed proteins. Thus, $p(k,k_{s})$ is the probability that a protein with a total of *k* links has exactly $k_{s}$ links to seed proteins and *p*-value $\left(k,k_{s}\right)$ is the probability that a protein with a total of *k* links has more connections to seed proteins than expected (Figure 2).
2. It ranks the proteins according to their respective *p*-values. The protein with the highest rank (i.e., lowest *p*-value) is called “candidate protein”.
3. It adds the candidate protein to the set of seed proteins, increasing their number from $s_{0}$ to $s_{1}=s_{0}+1$.
4. It iterates steps 1–3 with the expanded set of seed proteins, pulling one protein at a time into the growing disease module.

The procedure 1–4 can be repeated until the module spans across the entire network. The order in which the proteins are being added to the module provide a ranking of all proteins reflecting relevance association to the disease.

## 3. miRNA-Mediated Interactions Network: A Competing Endogenous RNA Model Exploiting the Topological Properties of Regulatory Networks

Recent findings have identified ceRNAs as the drivers in many disease conditions, including cancers [22,36,37,38,39]. They indirectly regulate each other by reducing the amount of miRNAs available to target messenger RNAs via the binding of MREs [40]. Recently, a computational method [25] was developed for identifying putative lncRNAs acting as miRNAs sponges in human breast cancer. In this study [25], the authors used normalized level three RNA- and miRNA- sequencing expression data of breast cancer adenocarcinoma (brca) from IlluminaHiSeq platform that were retrieved from TCGA (The Cancer Genome Atlas) [41,42]. The study concerned 72 samples for which the complete sets of tumor and matched normal profiles (for both RNA-seq and miRNA-seq data) were available. The computational ceRNA model is based on three hypotheses:

- RNAs competing for the same miRNA are marked by a highly positive correlation.
- Interaction between the RNAs competing for the same miRNA is indirect, i.e., mediated by miRNA.
- RNAs competing for the same miRNA harbor one or more MREs for the miRNA they sponge.

**Figure 3:**
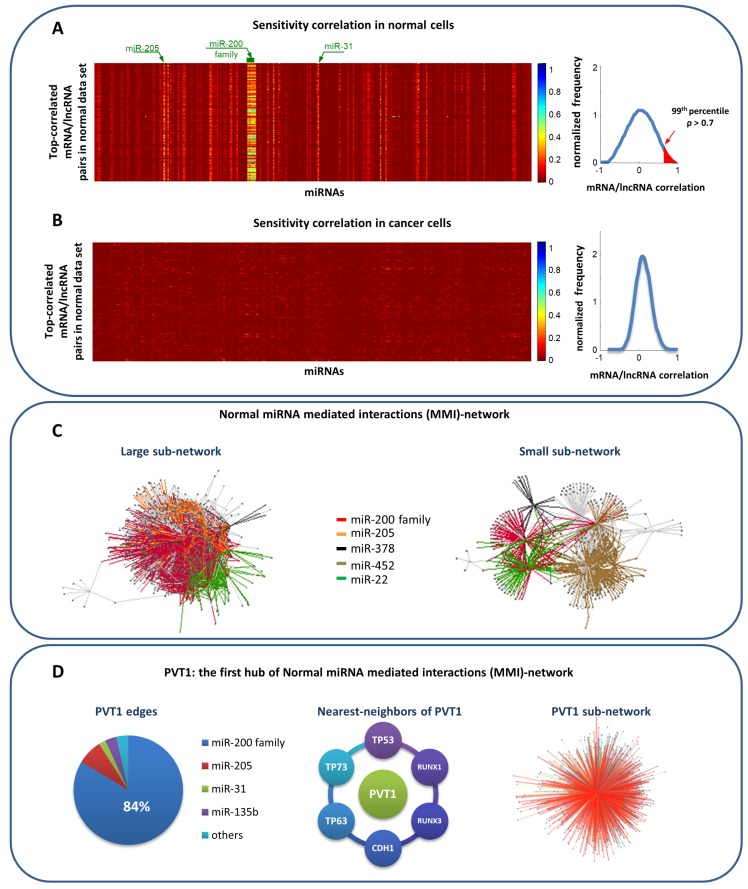
Results of Paci et al. model to predict miRNA sponge interactions in breast invasive carcinoma [25]. (**A**) Heatmap showing the sensitivity correlation for the top-correlated mRNA/lncRNA pairs (e.g., pairs for which the Pearson correlation between their expression profiles exceeds the 99th percentile of the overall correlation distribution) in normal breast tissues. Red color corresponds to zero sensitivity correlation, meaning that the interaction between the selected RNA pairs is direct and not mediated by miRNAs. Light vertical stripes point to miRNAs that are mediating the interaction, suggesting putative competing endogenous RNAs. (**B**) The same as in panel (**A**) but using data from breast cancer tissues. (**C**) The normal MMI-network (1738 nodes and 32,375 edges) built starting from the expression data of normal breast tissues. Nodes represent both mRNAs and lncRNAs; edges represent miRNAs that are mediating their interactions. Each pair of linked nodes fulfills two requirements: (i) sensitivity correlation > 0.3 and (ii) one or more shared MREs, for each miRNA linking them. Colors correspond to different miRNAs. (**D**) PVT1 subnetwork analysis. From left to right: the percentage of the miRNAs sponged by PVT1 with respect to all of its; some nearest neighbors of PVT1 that are well-known cancer genes; the sponge interactions sub-network of PVT1 (753 nodes and 2169 edges).

For what concerns the first hypothesis, the top-correlated messenger RNA (mRNA)/lncRNA pairs in normal and cancer data sets were selected by setting in both cases the correlation threshold to the 99th percentile of the corresponding overall correlation distribution (Figure 3A right).

For what concerns the second hypothesis, to investigate the scenario in which specific miRNAs may mediate the interactions of the top-correlated mRNA/lncRNA pairs, a well-established tool of multivariate analysis (i.e., the partial correlation) was applied to each selected mRNA/lncRNA pair with respect to each miRNA in their dataset. In general, the partial correlation ($\rho_{XY|Z}$) measures the extent to which an observed correlation between two variables X and Y (here, the expression profiles of a mRNA and a lncRNA) relies on the presence of a third controlling variable Z (here, the expression profile of a miRNA) and it is computed as:

(3) $$\rho_{XY|Z}=\frac{\rho_{XY}-\rho_{XZ}\rho_{ZY}}{\sqrt{1-\rho_{XZ}^{2}}\sqrt{1-\rho_{ZY}^{2}}}$$

where $\rho_{XY}$ is the Pearson correlation. Then, for each triplet mRNA/lncRNA/miRNA the *sensitivity correlation* *S*, defined as:

(4) $$S=\rho_{XY}-\rho_{XY|Z}$$

was computed. The XYZ triplets with S >0.3, corresponding to a drop of about the 30% in the correlation between XY when Z is removed, were selected. The sensitivity distribution of the top-correlated mRNA/lncRNA pairs (XY) is plotted removing one miRNA (Z) molecule at time (Figure 3A left).

For what concerns the third hypothesis, the triplets mRNA/lncRNA/miRNA that are enriched in binding sites of the shared miRNA (hypergeometric test *p*-value < 0.01) were selected by performing a seed match analysis. The minimal pairing requirement to predict a miRNA target recognition is a perfect match to positions 2–7 (6-mer miRNA seed) at the 5’-end of the mature miRNA sequence [43].

Integrating the results of multivariate statistical analysis and seed match analysis, the so-called miRNA-mediated interactions (MMI) network was built both in normal (Figure 3C) and cancer tissues [25]. Nodes in the networks represent mRNAs and lncRNAs with highly correlated expression profiles while edges represent miRNAs mediating their interactions. Normal MMI-network accounted for 1738 nodes and 32,375 edges (Figure 3C). Linked nodes are required to fulfill the above-mentioned hypothesis of the ceRNA model, which corresponds to the following mathematical constraints:

- Matching high values of the Pearson correlation between their expression profiles (ρ> 0.7);
- Matching high values of the sensitivity correlation (S >0.3);
- Sharing binding sites for miRNAs (6-mer miRNA seed match).

This study revealed the existence of a complex regulatory network in normal samples that appears to be missing in tumor samples (and vice-versa), highlighting a marked rewiring in the ceRNA program between normal and pathological breast tissues (Figure 3B). At the heart of this phenomenon, there was the recently and widely studied oncogene PVT1 [44,45,46,47,48,49,50,51,52,53,54,55,56,57,58,59,60,61,62,63] that switched from being the first of the hubs in the normal MMI network to fall outside the list of nodes of the cancer network. In normal network, PVT1 revealed a net binding preference towards the miR-200 family (Figure 3D), which antagonized to regulate the expression of hundreds of mRNAs that are known to be related to the cancer development and progression (e.g., GATA3, CDH1, TP53, TP63, TP73, RUNX1, and RUNX3). Despite its up-regulation in breast cancer tissues, mimicked by the miR-200 family members, PVT1 stopped working as ceRNA in the cancerous state.

**Figure 4:**
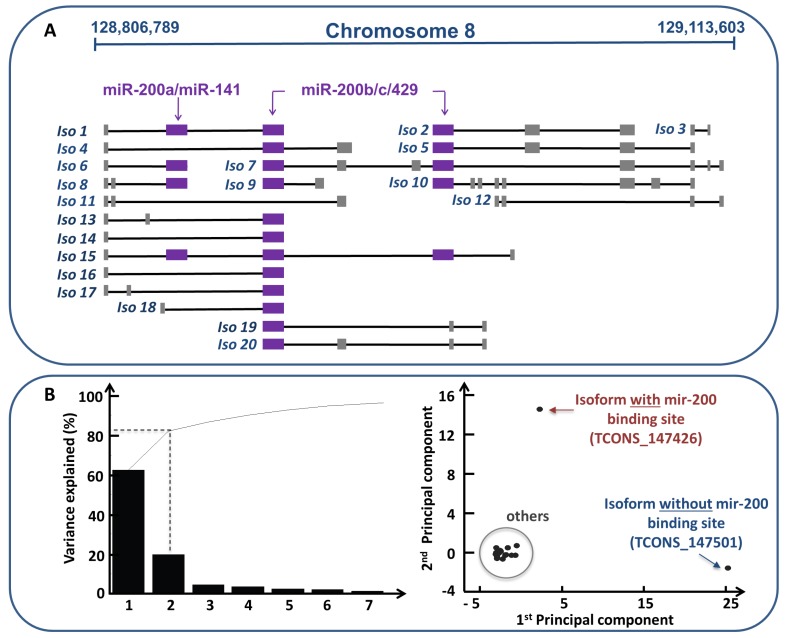

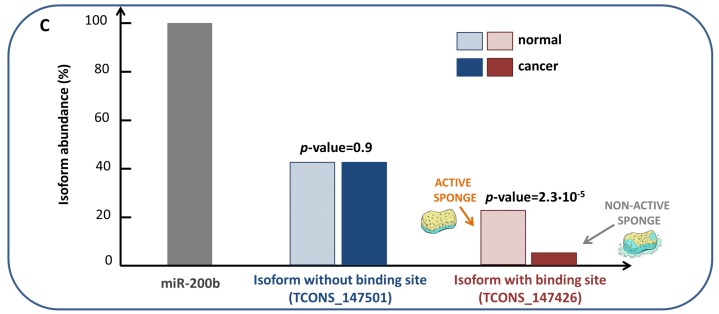
Analysis of PVT1 isoforms [27]. (**A**) Sketch of PVT1 genomic locus as reconstructed by Cufflinks spans across a genome interval of over 300 kb (i.e., 128,806,789-129,113,603 bases within the February 2009 human genome build GRCh37/hg19) on the forward strand of chromosome 8. PVT1 locus gives rise to 91 different variants according to raw RNA-seq data of TCGA (The Cancer Genome Atlas) for breast invasive carcinoma. The isoform names correspond to an increasing symbolic numbering and not to the actual nomenclature of the PVT1 variants. Lines represent introns and boxes (violet and grey) represent exons. Violet boxes correspond to the binding sites for the miR-200 family members. Note that some isoforms lack such binding sites (e.g., Iso11 and Iso12). (**B**) (Left) The percent variability explained by each principal component (PC) shown by the Pareto chart. This chart contains both bars and a line graph, where individual values are represented in descending order by bars, and the line represents the cumulative total value. The y-axis represents the percentage of the data variance explained by each PC, whereas the x-axis represents the principal components that are able to explain the first 100% of the cumulative distribution. PCA is performed using the variations of all the isoforms between normal and cancer tissues. (Right) The scatter plot of the projection of the original data (i.e., the variations of all the isoforms between normal and cancer tissues) onto the first two PCs; the x-axis contains the first PC while the y-axis contains the second PC. In this plot, it is possible to group isoforms in three classes: the isoform missing the binding site for the miR-200 family members (blue isoform, TCONS_147501), the isoform with the seed match for the miR-200b/200c/429 cluster (red isoform, TCONS_147426), and all the others. The first PC is able to separate the variation of the blue isoform from the others; the second PC is able to separate the variation of the red isoform from the others. (**C**) The ratio between the abundance of the red isoform (TCONS_147426, with the binding site for the miR-200b/200c/429 cluster) and blue isoform (TCONS_147501, without the binding site) with respect to the miR-200b in both normal (striped rectangle) and cancer tissues (full boxes). In the normal tissues only the isoform of PVT1 gene harboring the binding site for the miR-200b/200c/429 cluster acts as a sponge regulator of the miR-200 family members. In cancer tissues, it stops working as a sponge since its concentration is much lower than the concentration of the miR-200 family members.

The specific conditions required for a ceRNA landscape to occur are still far from being determined. However, in a recent study [27], the authors emphasized the importance of the relative concentration of the ceRNAs, and their related miRNAs. In particular, they focused on the withdrawal of the PVT1 ceRNA activity functioning as miR-200 sponge in breast cancer tissues, betting on a titration mechanism as the main culprit (i.e., large changes in the ceRNA expression levels either overcome, or relieve, the miRNA repression on competing RNAs; similarly, a very large miRNA over-expression may abolish competition). Firstly, they performed a gene expression and sequence analysis of PVT1 genomic locus, which revealed the existence of multiple isoforms representing all the possible configurations (Figure 4A): missing the binding site (e.g., Iso11 and Iso12 in Figure 4A); hosting the binding site for all (e.g., Iso1 in Figure 4A) or some members of the miR-200 family (e.g., Iso6 or Iso7 in Figure 4A). By performing the principal component analysis (PCA) using the feature abundance levels of all the PVT1 isoforms across normal and cancer samples, the authors found that two principal components (PCs) were able to explain more than the 80% of the variance of the data (Figure 4B left). In particular, the first PC–explaining about the 60% of the total variance of the analyzed data—referred to the variation of the TCONS_147501 isoform that, missing the binding site, did not interact with the miR-200 family; while the second PC—explaining by alone about the 20% of the total variance—corresponded to the variation of the TCONS_147426 isoform that, hosting the binding site for the miR-200b/200c/429 cluster, could be act as competitor of the targets of these miRNAs. By drawing the score plot (Figure 4B right), it emerged that the first PC and the second PC were able to to separate the contribution of the isoform missing the binding site for any members of the miR-200 family (i.e., TCONS_147501, blue isoform in Figure 4B) and of the isoform hosting the binding site for the miR-200b/200c/429 cluster (i.e., TCONS_147426, red isoform in Figure 4B) from all the others, respectively. Then, since the isoform harboring the binding site (i.e., TCONS_147426) and the isoform missing the binding site for the miR-200 family members (i.e., TCONS_147501) were the only isoforms that changed, the author evaluated the ratio between the abundance of each one with respect to one representative member of the miR-200b/200c/429 cluster (i.e., miR-200b) in both normal and cancer tissues (Figure 4C). From this analysis, they found that only the PVT1 isoform harboring the binding site for miR-200b showed a drastic decrease in its relative concentration with respect to the miRNA abundance from normal to cancer tissues, providing a plausibility argument to the breakdown of the sponge program orchestrated by the oncogene PVT1 (Figure 4C).

## 4. SWItchMiner (SWIM): A Tool Exploiting the Topological Properties of Gene Co-Expression Networks

SWItchMiner (SWIM) [28] is a wizard-like software implementation of a network-based model. By combining topological properties of co-expression networks with genome-wide analysis, SWIM is able to identify a small pool of genes, called switch genes that are critically associated with drastic changes in cell phenotype.

### 4.1. SWIM Algorithm

**Figure 5:**
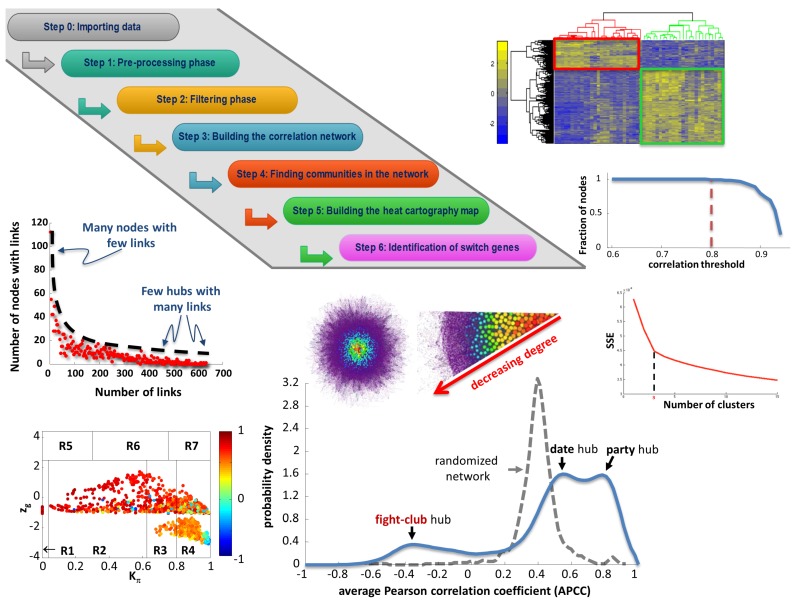
SWIM flowchart. The figure depicts the steps performed by SWIM [28] and shows some examples of outputs obtained by running SWIM on the grapevine dataset [29].

The algorithm implemented by SWIM [28] is composed of the steps depicted in Figure 5.

#### 4.1.1. Differential Gene Expression Analysis

**Pre-processing phase**. SWIM includes a pre-processing phase (Step 1 in Figure 5) for removing genes whose expression is mostly zero or change very little. This step requires the selection of two specific thresholds: the first threshold regards the maximum number of zeros allowed for the expression values of each gene across samples; the second one concerns the minimum variation—measured by the Inter Quartile Range (IQR) percentile—allowed for each gene across samples.

**Filtering phase**. SWIM implements a filtering phase (Step 2 in Figure 5) that allows to remove genes whose expression between two given conditions (A and B) does not change enough or does it without statistical significance. This step requires the selection of other two specific thresholds. Considering the logarithm of the ratio between the average expression of samples in condition A and the average expression of samples in condition B (log fold-change), the first threshold allows to remove the genes falling behind, in absolute value, a fixed cutoff on the log fold-change. The second threshold concerns the smallest probability (*p*-value) for which the data allow to reject the null hypothesis (i.e., the means of the two distributions are identical) of the Student’s *t*-test. Actually, since this statistical test will be repeated multiple times (as much as the genes under testing), the obtained *p*-values must be adjusted. To correct multiple tests, SWIM makes use of False Discovery Rate (FDR) method [64] and thus the threshold refers to the FDR values. At the end of this phase, the differentially expressed genes between conditions A and B have been identified.

#### 4.1.2. Network Analysis

**Building the correlation network**. SWIM builds a co-expression network of differentially expressed RNAs based on the Pearson correlation between the expression profiles of gene pairs (Step 3 in Figure 5). In this network, two nodes are connected if the absolute value of the Pearson correlation for their expression profiles is greater than a given threshold. The choice of this threshold should reflect a right balance between the number of edges and the number of connected components of the network: the number of edges should be as small as possible in order to have a manageable network (pointing towards a higher threshold) and the number of connected components should be as small as possible in order to preserve the integrity of the network (pointing towards a smaller threshold).

**Finding communities in the network**. To find communities in the network, SWIM makes use of the k-means algorithm [65], a method of cluster decomposition whose aim is to partition *n* objects (i.e., the nodes of the co-expression network) into *N* clusters (Step 4 in Figure 5). The quality of clustering was evaluated by minimizing the sum of the squared error (SSE), depending on the distance of each object to its closest centroid. A reasonable choice of the number of clusters is suggested by the position of an elbow in the SSE plot computed as a function of *N*. As distance measure, it is used dist(x,y)=1−ρ(x,y), where ρ(x,y) is the Pearson correlation between expression profiles of nodes *x* and *y*. The k-means algorithm, despite being the most widely used clustering algorithm, has some intrinsic limitations. Firstly, the number of clusters must be set in advance; secondly, it guarantees convergence only to a local minimum of SSE; thirdly, the initial position of the centroids is randomly chosen to cause a dependence of the partitioning on initialization. However, some reasonable assumptions can be done and are described in the following. There is no strict method to determine the “correct” number of clusters. Among others, SWIM uses an approach—named “Scree plot”—that evaluates the behavior of the SSE function to vary the number of clusters. Then, the position of an elbow in the scree plot—i.e., where the “cliff” reaches a bottom plateau—determines an appropriate number of clusters. Since finding the global optimum of SSE is theoretically NP-hard [66], it is commonly assumed that is sufficient to carry out a number of random initialization followed by a selection of the best separated solution, measured by the lowest SSE [67]. Moreover, the partition with the lowest SSE is commonly assumed to be reproducible under repeated initializations [67]. Thus, for a given number of clusters, SWIM allows repeating the clustering many times (replicates), each with a new set of initial cluster centroid positions. For each replicate, the k-means algorithm performs iterative partitioning (iterations) until the minimum of the SSE function is reached. Then, the cluster configuration with the lowest SSE values among all replicates will be chosen, for that number of clusters.

#### 4.1.3. Role assignment to network nodes

**Building the heat cartography map**. Once the modular structure of the complex network has been found, roles have to be assigned to each node. This is done by dividing the plan according to two parameters, the clusterphobic coefficient $K_{\pi }$ and the global within-module degree $z_{g}$. The clusterphobic coefficient $K_{\pi }$ measures the “fear” of being confined in a cluster, in analogy with the claustrophobic disorder. A high value of $K_{\pi }$ denotes nodes having much more external than internal links. The global within-module degree $z_{g}$ measures how “well-connected” each node is to other nodes in its own community. In the following, the formal definitions of these parameters for a generic node *i* [28]:

(5) $$K_{\pi }^{i}=1-{\left(\frac{k_{i}^{in}}{k_{i}}\right)}^{2}$$

(6) $$z_{g}^{i}=\frac{k_{i}^{in}-{\bar{k}}_{C_{i}}}{\sigma_{C_{i}}}$$

where $k_{i}^{in}$ is the number of links of node *i* to nodes in its module $C_{i}$, $k_{i}$ is the total degree of node *i*, ${\bar{k}}_{C_{i}}$ and $\sigma_{C_{i}}$ are the average and standard deviation of the total degree distribution of the nodes in the module $C_{i}$. This definition of $z_{g}$ quantifies how much a node is a hub (i.e., degree exceeding 5 [68]) in its community and thus represents a measure of local connectivity. On the contrary, the parameter $K_{\pi }$ evaluating the ratio of internal to external connections of a node represents a measure of global connectivity. Note that $K_{\pi }=0$ when a node has only links within its module, i.e., it does not communicate with the other modules ($k_{i}^{in}=k_{i}$); while $K_{\pi }$ is close to 1 when the majority of its links are external to its own module. According to the global within-module degree $z_{g}$ and the clusterphobic coefficient $K_{\pi }$ values, the plane is divided into seven regions (R1–R7), each defines a specific node role [69]. High $z_{g}$ values correspond to nodes that are hubs within their module (local hubs), while high values of $K_{\pi }$ identify nodes that interact mainly outside their community. Then, SWIM colors nodes in the cartography according to the average Pearson correlation coefficient (APCC) between the expression profiles of each node and its nearest neighbors [68]. This representation of the network is defined as “heat cartography map” (Step 5 in Figure 5). By computing the APCC of expression over all interaction partners of each hub in PPI networks in yeast, the authors in [68] concluded that hubs fall into two distinct categories: date hubs that display low co-expression with their partners (low APCC) and party hubs that have high co-expression (high APCC). In the gene expression networks, the distribution of APCCs appears to be trimodal [28,29] where, similar to PPI networks, two peaks represent low (date hubs) and high (party hubs) positive APCC values, but with the addition of a new third peak which is characteristic of gene expression networks and represents negative APCC values. Nodes populating this peak are called “fight-club hubs” [29].

**Identification of switch genes**. Looking at the heat cartography map, SWIM identifies the so-called “switch genes” (Step 6 in Figure 5): the subset of the fight-club hubs that mainly interact outside their community (region R4). In particular, they satisfy the following topological and expression features:

- Being not a hub in their own cluster ($z_{g}<2.5$);
- Having many links outside their own cluster ($K_{\pi }>0.8$);
- Having a negative average weight of their incident links (APCC <0).

At the end of Step 6, SWIM gives the opportunity to perform further analyses regarding the evaluation of network robustness, which is the resilience to errors, by studying the effect on the network connectivity of removing nodes by decreasing degree. In particular, SWIM evaluates the effect on the average shortest path (the shortest path between two nodes is the minimum number of edges connecting them and the average shortest path of a network is the average of the shortest paths for all possible pairs of network nodes) of removing randomly chosen nodes, switch genes, fight-club hubs, date and party hubs. Since scale-free networks have few hubs and many non-hub nodes, they are amazingly resistant to a random removal of nodes, while the removal of hubs causes an effect known as “vulnerability to attack” to allude to the fact that the integrity of the network is destroyed.

### 4.2. SWIM Applications

SWIM was amenable to detect switch genes in different organisms and cell conditions, leading to the identification of key players in biologically relevant scenarios, including but not limited to human cancer [28,29,30].

#### 4.2.1. Grapevine Analysis

**Figure 6:**
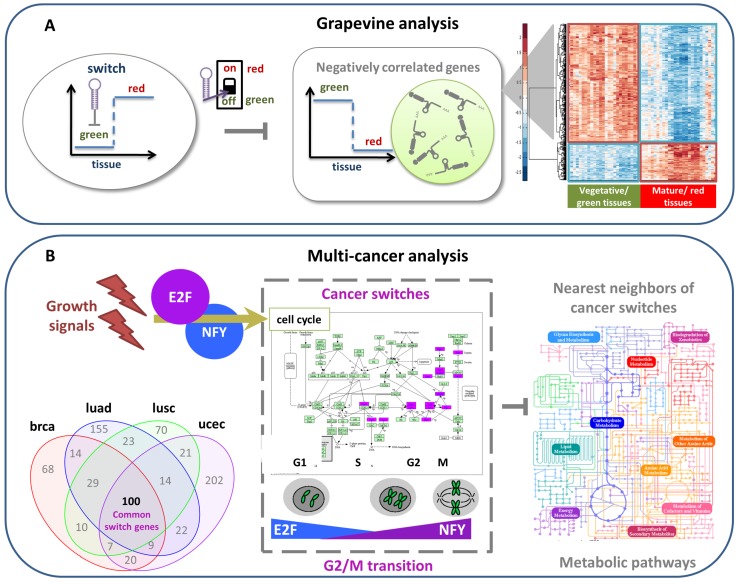

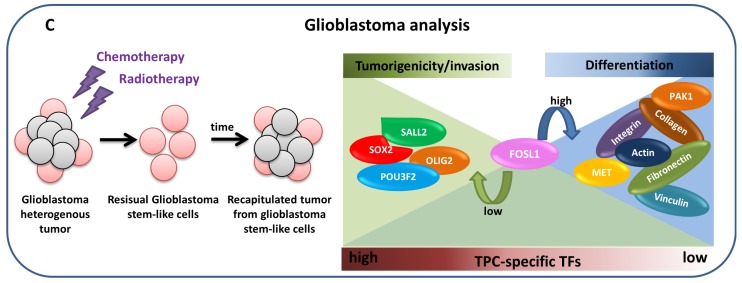
SWIM applications in **(A**) grapevine analysis [29], (**B**) multi-cancers analysis [28], and (**C**) glioblastoma analysis [30]. (**A**) A sketch of the switch gene regulation mechanism in grapevine. During the vegetative/green phase of organ development, switch genes are repressed by miRNAs and vegetative genes are expressed. In the transition to the mature/red phase, these miRNAs are deactivated, the switch genes are expressed and their anti-correlated vegetative genes are turned off. The heat map shows the transcription level of positively and negatively correlated genes with a typical switch, where expression values increase from blue to red. (**B**) A sketch of the switch gene regulation mechanism in human cancers. SWIM extracted a set of 100 cancer-recurrent switch genes across four tumors—breast invasive carcinoma (brca), lung squamous cell carcinoma (lusc), lung adenocarcinoma (luad), uterine corpus endometrial carcinoma (ucec)—that showed a marked functional enrichment in cell cycle and specifically on the regulation of the G2-to-M transition. The promoter motif analysis suggested that two major transcription factors (namely E2F and NFY) lead to the activation of the switch gene layer of gene regulation. Activation of switch genes in these cancers seems to predominantly repress several metabolic pathways, possibly leading to the well-known metabolic rewiring characterizing cancer cells. (**C**) A sketch of the switch gene regulation mechanism in human glioblastoma. Glioblastoma subpopulation of self-renewing, stem-like cells has been shown to be responsible for tumor initiation, progression, resistance to treatment, and relapse. Among switch genes identified by SWIM involved in the transition from a stem-like to a differentiated phenotype of glioblastoma cells, FOSL1 stands out as a promising candidate to trigger the differentiation. On one hand, it has been found positively correlated with genes encoding proteins linked to the focal adhesion complex and extracellular matrix (ECM) receptor interaction (e.g., integrins, collagen, and signaling proteins). Conversely, it is negatively regulated with well-known neurodevelopmental transcription factors (TFs) specific of stem-like identity, including the core set of OLIG2, POU3F2, SALL2, SOX2 [70]. Thus, it could be considered as putative controller of stem-like cell differentiation process by repressing the core set of neurodevelopmental TFs and by modulating the equilibrium between cell adhesion and migration.

SWIM was successfully applied in plants [29] for studying the transition between mature to immature phase of the developmental program of of *Vitis Vinifera*. In this study, switch genes resulted to be master regulators of the transcriptome remodeling that marks the developmental shift of grapevine from immature to mature growth. Specifically, the authors found about one hundred switch genes in grapevine that appears to be anti-correlated with about 1000 genes (more than 50% of the entire co-expression network). All switch genes, expressed at low levels in vegetative/green tissues, showed a significant increase in mature/woody organs, suggesting a potential regulatory role in the immature-mature transition. Among switch genes, they found many transcription factors like NAC-domain genes and many targets of tissue-specific miRNAs, such as the NAC33 switch gene and miRNA-164 whose interaction was experimentally validated. The authors propose a transcriptional regulatory network in which tissue-specific and stage-specific miRNAs regulate the expression of several switch genes (Figure 6A). Switch genes appeared to be over-expressed in mature organs and they are presumably regulated by tissue specific microRNAs with an opposite trend. They resulted also anti-correlated with thousands of genes that were down-regulated in mature tissue, meaning that the transition to mature growth in grapevine was mainly due to the suppression of vegetative pathways such as photosynthesis and cell proliferation rather than the activation of maturation-specific pathways. The immature fruits grow and make photosynthesis, then stop growing and start ripening and activate the secondary metabolism like color, aroma and flavor of the fruit. The results of this study are very important for the winemaking industries because the identification of which genes are responsible for the ripening can help to face climate changes and thus improve the quality of wine.

#### 4.2.2. Multi-Cancer Analysis

SWIM was applied to a large panel of cancer datasets obtained from TCGA [41,42] to identify switch genes that could be critically associated with the drastic changes in the physiological state of tissues induced by the cancer development [28]. In this study, the authors found disease-specific switch genes, as well as common switch genes that were shared among different tumors. The list of common switch genes encompassed 100 RNA transcripts that appeared all up-regulated in cancer and enriched in cell cycle, in particular at the transition between the G2 phase and M phase. Moreover, their promoter regions appeared enriched in three known regulatory motifs: the cell cycle gene homology (CHR) element, the nuclear transcription factor Y (NFY) binding motif the E2F transcription factor (E2F) binding motif, that are known to participate in the regulation of progression through the cell cycle. Finally, the analysis of the functional annotation of their negative nearest neighbor highlighted a general association of crucial nodes of metabolic process. The authors propose a regulatory network to describe the switch gene mechanism in human cancers (Figure 6B). In summary, the list of 100 common switch genes appear to be over-expressed in cancer tissues, suggesting a their potential role in the malignant transformation; they are involved in cell cycle, at the G2/M transition; they are anti-correlated to some metabolic pathways that in turn appear to be switched-off in cancer and they are activated by growth signals. The activation of switch genes by growth transcription factors could accelerate the late phase of the cell cycle with a consequent increase of cancer progression and promote the rewiring of some metabolic pathways, hallmarks of the malignant transformation [71].

The list of disease-specific switch genes identified by SWIM encompassed protein coding genes, long non-coding, and miRNAs, recovering many known key cancer players, but also many new potential biomarkers not yet characterized in cancer context. Motivated by the growing interest in lncRNAs, which appears to hold strong promise as novel biomarkers and therapeutic targets for cancer [72,73], and by the widespread recognized role of miRNAs as key negative regulators in many intracellular processes as well as in carcinogenesis [74], in the following we discuss miRNAs, lncRNAs, and mRNAs acting as switch genes in the multi-cancer analysis across TCGA cancer datasets, separately.

**Figure 7:**
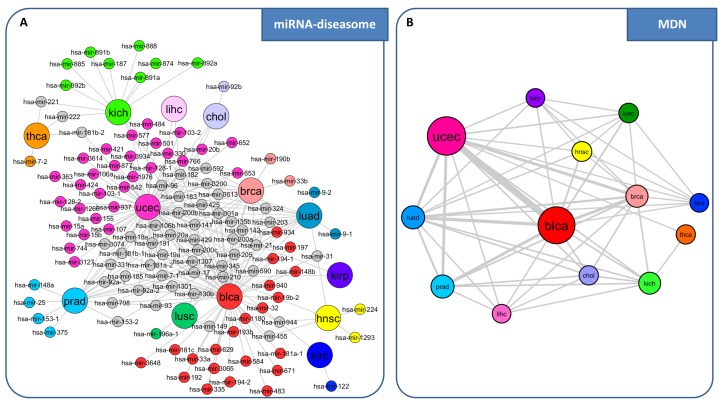
Comparative analysis of miRNAs acting as switch genes in the large panel of TCGA cancer datasets. (**A**) miRNA-diseasome. The bipartite network is composed of two disjoint sets of nodes with different size: the larger ones correspond to the analyzed human cancer types from TCGA, whereas the smaller ones correspond to all miRNAs acting as switch genes. A link occurs between a tumor type and a miRNA if the miRNA acts as switch gene for that tumor. Different colors are associated to different tumor types. miRNAs are colored based on the tumor type to which they belong. Nodes are light gray if the corresponding miRNAs are associated with more than one tumor type. (**B**) miRNA-disease network (MDN). The MDN is the projection of the miRNA-diseasome bipartite network, in which nodes correspond to tumor types (diseases) and two diseases are connected if there is at least one miRNA that acts as switch gene in both. The width of a link is proportional to the number of miRNAs that are acting as switch genes in both diseases. The size of a node is proportional to the number of microRNAs acting as switch genes for that disease. Different node colors are associated with different diseases. blca: bladder urothelial carcinoma, chol: cholangiocarcinoma, hnsc: head and neck squamous cell carcinoma, kich: kidney chromophobe, kirc: kidney renal clear cell carcinoma, kirp: kidney renal papillary cell carcinoma, lihc: liver hepatocellular carcinoma, prad: prostate adenocarcinoma, thca: thyroid carcinoma.

**miRNA-diseasome network**. We built a diseasome bipartite network consisting of two disjoint sets of nodes: one set corresponds to the human cancer types under study (diseases); the other set corresponds to all miRNAs acting as switch genes in each disease (Figure 7A). A disease and a miRNA are then connected by a link if that miRNA acts as switch gene in that disease. This representation allows to highlight which are miRNAs involved in multiple diseases (i.e., grey nodes in Figure 7A) and which are disease-specific miRNAs for each tumor (i.e., nodes that are colored according to each tumor type in Figure 7A). We called this network “miRNA-diseasome”, in analogy with the human diseasome network of [2], where the two disjoints set of nodes corresponds to disorders and disease genes. In this diseasome bipartite network a link is placed between a disorders and a disease gene if mutations in that gene lead to the specific disorder. Then, starting from the miRNA-diseasome bipartite network, we generated a biologically relevant network projection that we called “miRNA-disease network” (MDN) (Figure 7B). In the MDN nodes represent diseases, and two diseases are connected to each other if they share at least one miRNA acting as switch gene in both diseases. This representation provides a disease-centered view of the miRNA-diseasome and allows us to highlight tumors with the highest number of miRNAs acting as switch genes (i.e., blca that results as a major hub in the network), as well as tumors with the highest number of shared miRNAs (i.e., blca and ucec).

**Figure 8:**
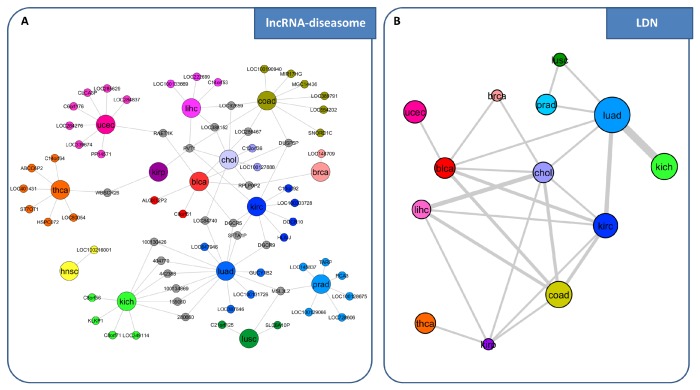
Comparative analysis of lncRNAs acting as switch genes in the large panel of TCGA cancer datasets. (**A**) lncRNA-diseasome. The bipartite network is composed of two disjoint sets of nodes with different size: the larger ones correspond to the analyzed human cancer types from TCGA, whereas the smaller ones correspond to all lncRNAs acting as switch genes. A link occurs between a tumor type and a lncRNA if the lncRNA acts as switch gene for that tumor. Different colors are associated to different tumor types. lncRNAs are colored based on the tumor type to which they belong. Nodes are light gray if the corresponding lncRNAs are associated with more than one tumor type. (**B**) lncRNA-disease network (LDN). The LDN is the projection of the lncRNA-diseasome bipartite network, in which nodes correspond to tumor types (diseases) and two diseases are connected if there is at least one lncRNA that acts as switch gene in both. The width of a link is proportional to the number of lncRNAs that are acting as switch genes in both diseases. The size of a node is proportional to the number of lncRNAs acting as switch genes for that disease. Different node colors are associated with different diseases.

**lncRNA-diseasome network.** We performed the same analysis as for miRNAs in order to build a lncRNA-diseasome bipartite network, where one set of nodes corresponds to the human cancer types analyzed by SWIM in [28] and the other set corresponds to all lncRNAs acting as switch genes in each disease (Figure 8A). A disease and a lncRNA are then connected by a link if that lncRNA acts as switch gene in that disease. Then, starting from the lncRNA-diseasome bipartite network, we generated the “lncRNA-disease network” (LDN) (Figure 8B), where nodes represent diseases, and two diseases are connected to each other if they share at least one lncRNA acting as switch genes in both diseases.

**Figure 9:**
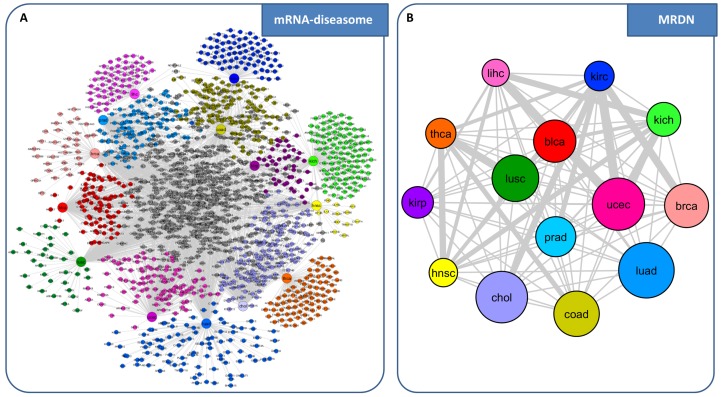
Comparative analysis of protein-coding genes (mRNAs) acting as switch genes in the large panel of TCGA cancer datasets. (**A**) mRNA-diseasome. The bipartite network is composed of two disjoint sets of nodes with different size: the larger ones correspond to the analyzed human cancer types from TCGA, whereas the smaller ones correspond to all protein coding genes acting as switch genes. A link occurs between a tumor type and a mRNA if the mRNA acts as switch gene for that tumor. Different colors are associated to different tumor types. mRNAs are colored based on the tumor type to which they belong. Nodes are light gray if the corresponding miRNAs are associated with more than one tumor type. (**B**) mRNA-disease network (MRDN). The MRDN is the projection of the mRNA-diseasome bipartite network, in which nodes correspond to tumor types (diseases) and two diseases are connected if there is at least one mRNA that acts as switch gene in both. The width of a link is proportional to the number of mRNAs that are acting as switch genes in both diseases. The size of a node is proportional to the number of mRNAs acting as switch genes for that disease. Different node colors are associated with different diseases.

**mRNA-diseasome network.** Finally, we built a mRNA-diseasome bipartite network, where one set of nodes corresponds to the human cancer types analyzed by SWIM in [28] and the other set corresponds to all mRNAs acting as switch genes in each disease (Figure 9A). Then, as before, starting from the mRNA-diseasome bipartite network, we generated the “mRNA-disease network” (MRDN) (Figure 9B), where nodes represent diseases, and two diseases are connected to each other if they share at least one mRNA acting as switch genes in both diseases.

#### 4.2.3. Glioblastoma analysis

Glioblastoma multiforme (GBM) is the most frequently diagnosed and aggressive brain tumor with the 5-years survival rate achieved for only 5% of patients. Several studies identified a subpopulation of GBM cells with radio/chemotherapy-resistant properties that have a role in driving tumor initiation, progression, resistance to treatment, and relapse [75]. Due to their abilities of self-renewal, proliferation, and differentiation into multiple lineages, these cells are named cancer stem-like cells and are held responsible for carcinogenesis (Figure 6C). The identification of genes responsible of the stem-like phenotype are going to dominate cancer research scene as effective and long-lasting therapeutic strategy. In this context, a recent study [70] identified a 4-core of neurodevelopmental TFs (transcription factors) (i.e., OLIG2, POU3F2, SALL2, SOX2), which are selectively expressed in glioblastoma stem-like cells and have been shown to be sufficient to fully reprogram differentiated cells into glioblastoma stem-like cells. In order to computationally identify genes controlling cancer stem-like cells differentiation and invasion, SWIM was applied to gene expression profiles from two independent GBM datasets [30], publicly available on the Gene Expression Omnibus (GEO) repository: RNA-seq data obtained from stem-like tumor-propagating cells and differentiated glioblastoma cells (i.e., GSE54792 [70]); Affymetrix HG-U133 Plus 2.0 microrarrays expression data from glioblastoma stem-like cell lines, the corresponding primary tumors, and conventional glioma cell lines (i.e., GSE23806 [76])).

SWIM identified the FOS like transcription factor FOSL1 as the most promising switch gene (Figure 6C) shared from both datasets [30]. Indeed, FOSL1 fulfills very interesting features that made it eligible as new potential therapeutic target: it is down-regulated in stem-like cells; it is highly negatively correlated with the 4-core TFs (OLIG2, POU3F2, SALL2, SOX2); the promoter regions of the 4-core TFs were found to harbor a consensus binding motif for FOSL1; it was found to act as repressor transcription factor [77]; it is positively correlated with genes encoding proteins crucial for cell-matrix adhesion and cell motility (e.g., actin, collagen, fribonectin, and several integrins), which can influence cell adhesion dynamics and migration, and thus the cancer invasiveness.

Taken together these considerations prompted the authors to bet on FOSL1, which could promote the differentiation process of GBM stem-like cells by repressing the 4-core TFs and consequently halted cancer growth and invasion. This should allow for anticipation of care as well as the reduction of the social impact of diseases and the restraint of health costs.

### 4.3. SWIM Switch Genes towards DIAMOnD Disease Genes

Next we explore the performance of SWIM on human breast invasive carcinoma. Since the full set of disease proteins is unknown, we cannot assess the performance directly in terms of true positives/negatives. We therefore compare the results obtained by SWIM with the known disease-associated genes and with the DIAMOnD disease genes. SWIM was applied to breast invasive carcinoma expression data from high-throughput RNA-seq downloaded from TCGA data portal. Data correspond to normalized level three data from RNASeq Version 2 created by using MapSplice [78] to do the alignment and RSEM [79] to perform the quantification and normalization. The study concerned 103 samples for which the complete sets of tumor and matched normal profiles were available. By running SWIM, we obtained 257 switch genes and we selected for this comparison only the 195 switch genes that are coding RNAs. The known disease-associated genes for breast neoplasms disease (in total $s_{0}=40$ seed proteins) were provided by DIAMOnD paper [10] that integrated data from OMIM (Online Mendelian Inheritance in Man) [80] and GWAS (Genome-Wide Association Studies). Finally, the DIAMOnD disease genes were retrieved by running DIAMOnD algorithm for breast neoplasms disease and retaining the first 500 new disease genes. In total, we considered 540 seed genes, including the 40 breast neoplasm associated genes and the first 500 DIAMOnD disease genes.

**Figure 10:**
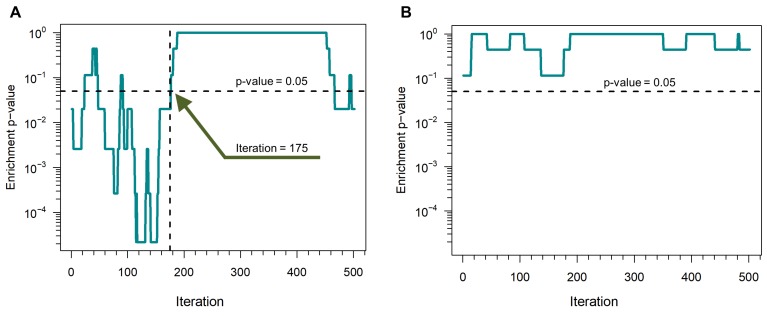
Performance of SWIM in human breast invasive carcinoma. (**A**) Enrichment *p*-values of switch genes in seed genes obtained by running SWIM for breast invasive carcinoma. (**B**) Enrichment *p*-values for a subset of genes drawn randomly from the network in seed genes.

We compared the performance of SWIM switch genes to seed genes as well as to random expectation for the same number of genes drawn randomly from the network. The performance is based on the number of switch genes that are considered true positives. To quantify the statistical significance of a given number of true positives at a given iteration step *i*, we used a sliding window approach: at each iteration step *i* the same number of seed genes was considered. We used genes in the interval [$s_{0}+\left(i-1\right)$] until the sliding window spans across the entire set of 540 seed genes and count the number of true positives among switch genes. The statistical significance of an observed number is then determined using the hypergeometric distribution. For breast neoplasms disease, we found that switch genes are significantly enriched (*p*-value <0.05) in seed genes until 175 iterations (Figure 10A), significantly higher than random expectation (Figure 10B). This results is extremely encouraging since the authors of DIAMOnD claim in the paper [10] that the first ∼200 DIAMOnD genes are found to participate in important seed pathways at a rate similar to the one within the seed proteins themselves.

## 5. Conclusions

In the last decade, the great advances in high-throughput technologies have led to massive amounts of genomic, transcriptomic, proteomic and metabolomic data capable to provide new opportunities for identifying potential biomarkers and developing effective treatments for human diseases. The availability of this huge amount of data has revolutionized biomedical science and, in particular, cancer genomics, but only the amount is not enough. If in the past there was a difficulty in collecting genetic data, today the challenge is to give them meaning and it is therefore essential to use effective informatics solutions capable of managing, analyzing and integrating these biological “Big Data”. The need to take on this new challenge has paved the way for a paradigm shift towards the development of temporal and spatial multi-level models, from molecular machineries to single cells, whole organism and individuals, including the environment, to reveal the underlying links between components. This new type of medical paradigm is called “Network Medicine”. Rather than trying to understand pathogenesis into a reductionist framework, network medicine entwines the many facets of disease in many different types of networks: from the physical interactions acting in a cell to the information flow through biological components. The representation of complex systems as networks is of paramount importance for visualizing the interactome underlying structure, revealing new functional roles, and proposing new and fresh interpretations of data. Networks can be obtained from any sort of information: known protein–protein interactions, gene expression profiles, functional annotation, etc. In this review, we focused on three different classes of approaches that use different types of interaction networks to infer novel cancer genes: methods using PPI networks, methods using regulatory networks and methods using co-expression networks.

One important bias in the methods that predict cancer genes is the direct or indirect incorporation of prior knowledge. Methods using PPI networks suffer more from this problem compared with methods using co-expression and/or regulatory networks. In fact, many proteins or genes have been extensively studied and hence have a higher number of connections in the protein networks. Moreover, since network biology is still far from completing the human interactome, PPI networks are suffering from false negatives (i.e., missing interactions) and false positives (i.e., false interactions) [1]. In the future, much computational effort is needed to complete the human interactome and increase its confidence.

Finally, network-based approaches using PPI networks are restricted to mutations that affect protein coding regions of the genome, thus they cannot be used to predict novel cancer genes among ncRNAs. Although the other two classes of approaches discussed in this review do not suffer of this bias, they lack the capability to integrate different data collections. In the future, we expect that the development of more accurate computational tools will be able to overcome this limitation.

This review provides a limited view of the landscape of the existing approaches that using network theory to deal with issues related to medicine, but it has the potential to stimulate the growth of new methods as well as the improvement of the existing ones. This will fuel advances in network medicine supporting the planning of disease prevention and treatment.

## Figures and Tables

**Table 1:** Overview of network-based approaches to medicine.

Method/Tool	Brief Description	Availability	Reference
**Protein–Protein Interaction Network**			
Oti et al.	It identifies new candidate disease genes by searching for disease proteins having interaction partners located within loci associated with the same disease	Prediction results available	[7]
GenePANDA (Gene Prioritizing Approach using Network Distance Analysis)	It identifies new candidate disease genes based on their relative distance to known disease genes in a functional association network	Prediction results available	[8]
DADA (Degree-Aware Disease Gene Prioritization)	It prioritizes candidate disease genes with respect to a disease of interest based on network proximity measure, calculated by using Random Walk with Restarts algorithm [32] with some statistical adjustment	MATLAB software package	[9]
DIAMOnD (DIseAse MOdule Detection)	It identifies full disease modules around a set of known disease proteins by performing a systematic analysis of the PPI-network that exploits the “connectivity significance” instead of local connection density	Python software package	[10]
PRINCE (PRIoritizatioN and Complex Elucidation)	It prioritizes genes related to a query disease based on their closeness, in the PPI-network, to genes causing phenotypically similar disorders to the query disease	Cytoscape Plug-in	[11,33]
ProDiGe (Prioritization Of Disease Genes)	It implements a novel machine learning strategy for gene prioritization based on learning from a set of positive examples (e.g., known disease genes) and unlabeled examples (e.g., candidate genes), allowing heterogeneous data integration	MATLAB software package	[12]
**Regulatory Network**			
MMI-network (MiRNA-Mediated Interactions network)	ceRNA model based on partial association to investigate the role of lncRNAs as miRNA sponges in human breast cancer. It computes for each triplet (lncRNA, miRNA, messenger RNA (mRNA)) the difference between Pearson correlation of (lncRNA, mRNA) and partial correlation (lncRNA, mRNA|miRNA) to examine the contribution of the miRNA into the lncRNA/mRNA relationship	Prediction results available	[25,27]
PANDA (Passing Attributes between Networks for Data Assimilation)	It implements a message-passing model using multiple sources of information to predict regulatory relationships, and used it to integrate protein–protein interaction, gene expression, and sequence motif data to reconstruct genome-wide, condition-specific regulatory networks in yeast as a model	MATLAB/ R/Python software packages	[34]
Sonawane et al.	It uses PANDA to infer gene regulatory networks for 38 different tissues by integrating GTEx RNA-sequencing (RNA-seq) data with a canonical set of transcription factors to target gene edges and protein–protein interactions	Prediction results available	[35]
**Co-Expression Network**			
SWIM (Switch Miner)	Wizard-like software that integrates gene expression data with network topological properties for identifying a small pool of genes (i.e., switch genes) critically associated with drastic changes in cell phenotype	MATLAB software package	[28,29,30]
WGCNA (Weighted Correlation Network Analysis)	Collection of R functions for performing weighted correlation network analysis of large data sets, including functions for network construction, module identification, topological properties calculation, data manipulation and visualization	R software package	[31]
